# Differential Impact of *TGFB1* Variation by Metastatic Status in Androgen-Deprivation Therapy for Prostate Cancer

**DOI:** 10.3389/fonc.2021.697955

**Published:** 2021-05-25

**Authors:** Masaki Shiota, Naohiro Fujimoto, Takashi Matsumoto, Shigehiro Tsukahara, Shohei Nagakawa, Shohei Ueda, Miho Ushijima, Eiji Kashiwagi, Ario Takeuchi, Junichi Inokuchi, Takeshi Uchiumi, Masatoshi Eto

**Affiliations:** ^1^ Department of Urology, Kyushu University, Fukuoka, Japan; ^2^ Department of Urology, School of Medicine, University of Occupational and Environmental Health, Kitakyushu, Japan; ^3^ Department of Clinical Chemistry and Laboratory Medicine, Graduate School of Medical Sciences, Kyushu University, Fukuoka, Japan

**Keywords:** androgen-deprivation therapy, metastasis, prostate cancer, SNP, TGFB1

## Abstract

Transforming growth factor-β1 (TGF-β1) plays a dual role in cancer, acting as a tumor suppressor in the early stage of cancer development and as a tumor promoter in the later stage of cancer progression in various cancers. In this study, we investigated the association between genetic polymorphisms in *TGFB1* and clinicopathological characteristics or oncological outcome in prostate cancer cases treated with androgen-deprivation therapy (ADT) according to metastasis status. Japanese male patients with hormone-sensitive prostate cancer treated with ADT from 1993 to 2005 were included in this study. Genomic DNA was obtained from whole blood samples, and genotyping of *TGFB1* (rs2241716 and rs4803455) was performed by PCR-based technique. No significant association between genetic polymorphisms in *TGFB1* (rs2241716 and rs4803455) and clinicopathological parameters or prognosis was observed in patients with non-metastatic disease. In patients with metastatic disease, Gleason score in CT/TT carriers (rs2241716) and CA/AA carriers (rs4803455) was unfavorable compared with CC carriers. In addition, the CT/TT alleles in rs2241716 (hazard ratio, 1.82; 95% confidence interval, 1.12–2.94; P = 0.015) and the CA/AA alleles in rs4803455 (hazard ratio, 1.75; 95% confidence interval, 1.03–2.98; P = 0.040) were associated with a higher risk of progression during ADT compared with the CC allele in patients with metastatic disease. *TGFB1* genetic variations were associated with adverse characteristics and progression risk in ADT among patients with metastatic disease, but not those with non-metastatic disease, supporting a distinct role of TGF-β signaling between non-metastatic and metastatic prostate cancer.

## Introduction

Although most prostate cancer cases primarily respond to androgen-deprivation therapy (ADT), most of them eventually progress to castration-resistant prostate cancer (CRPC) (1). The aberrant activation of androgen receptor (AR) signaling, despite low levels of serum androgen, has been revealed to be critical in the progression to CRPC (2). Recently, intensive up-front therapies using docetaxel or novel AR-pathway inhibitors for metastatic hormone-sensitive prostate cancer have been proven to prolong survival and become standard therapy (3–5). However, although several risk models have been developed to estimate patient prognosis, it has been difficult to precisely predict the survival (3, 4, 6, 7).

Metastasis is the critical step for cancer progression, and the major cause of cancer-related mortality (8). Epithelial-mesenchymal transition (EMT) of cancer cells, which involves morphological and functional changes, is required for cells to metastasize to distant regions (9). Transforming growth factor-β1 (TGF-β1) is a pleiotropic polypeptide that forms multimeric complexes with two type I and two type II receptors and regulates various cellular functions such as differentiation, cellular proliferation, survival, apoptosis, migration, adhesion, angiogenesis, and immune surveillance (10). TGF-β1 has been shown to play a dual role in cancer, acting as a tumor suppressor in the early stage of cancer development and as a tumor promoter in the later stage of various cancers including prostate cancer (11). TGF-β signaling also interacts with EMT as well as AR signaling in prostate cancer, which may affect the therapeutic effect of ADT (12–15). Several studies have reported an association of genetic polymorphisms in *TGFB1*, which encodes TGF-β1, with cancer phenotypes in prostate cancer (16–19). Together, these findings suggest that genetic polymorphisms in *TGFB1* may be associated with cancer phenotypes in the early and later stages.

In this study, we investigated the association between genetic polymorphisms in *TGFB1* and clinicopathological characteristics or oncological outcomes in patients with prostate cancer during ADT by cancer stage.

## Patients and Methods

### Patients

This study included Japanese patients with non-metastatic prostate cancer treated with primary ADT or salvage ADT for prostate-specific antigen (PSA) recurrence after definitive therapy with radical prostatectomy or radiotherapy to prostate (non-metastatic disease) as well as patients with *de novo* metastatic prostate cancer to distant sites treated with primary ADT (metastatic disease) at the University of Occupational and Environmental Health (Kitakyushu, Japan) and Kyushu University Hospital (Fukuoka, Japan) from 1993 to 2005, as described previously (20–22).

Clinical TNM staging was determined in accordance with the unified TNM criteria based on the results of digital rectal examination, transrectal ultrasound, magnetic resonance imaging, computed tomography, and bone scan (23). ADT was performed with surgical castration or continuous medical castration using a gonadotropin-releasing hormone agonist (goserelin acetate or leuprorelin acetate) and/or an antiandrogen agent (bicalutamide, flutamide, or chlormadinone acetate). Progressive disease was defined as an increase in serum PSA levels >2 ng/mL and a 25% increase over the nadir, the appearance of a new lesion, or the progression of one or more known lesions classified according to the Response Evaluation Criteria in Solid Tumors (24).

Written informed consent was obtained from all patients. Patients who chose not to participate in this study were excluded. This study was performed in accordance with the principles described in the Declaration of Helsinki and the Ethical Guidelines for Epidemiological Research enacted by the Japanese Government and was approved by each institutional review board.

### Genotyping

Genomic DNA was extracted from whole blood samples from patients as previously described (20–22). Rs2241716 and rs4803455 were selected as representative single nucleotide polymorphisms of the *TGFB1* gene as described previously (20). Minimum minor allele frequency was set as 0.05 according to the HapMap database (http://hapmap.ncbi.nlm.nih.gov/index.html). Linkage disequilibrium analysis was performed with HaploView and the minimum r^2^ threshold was set as 0.8. Genotyping of *TGFB1* (rs2241716 and rs4803455) was performed on a CFX Connect Real-Time System (Bio-Rad, Hercules, CA, USA) with pre-designed TaqMan SNP Genotyping Assays (C_15873887_10 and C_30031638_10, respectively; Life Technologies, Carlsbad, CA, USA) and TaqMan Gene Expression Master Mix (Life Technologies), according to the manufacturers’ protocols.

### Statistical Analyses

All statistical analyses were performed using JMP14 software (SAS Institute, Cary, NC, USA). Categorical and continuous data were analyzed by Pearson’s chi-square and Wilcoxon rank sum tests, respectively. Survival analyses were conducted using the Kaplan–Meier method and the log-rank test. Univariate and multivariate analyses were performed using the Cox hazard proportional model to estimate hazard ratios (HRs). The differential prognostic value of *TGFB1* genotype was investigated through interaction tests. All *P*-values were two-sided. *P*-values < 0.05 were considered significant.

## Results

The clinical and pathological characteristics of the 101 prostate cancer patients with non-metastatic disease and 93 prostate cancer patients with metastatic disease included in this study are listed in **Tables 1** and **2**. In patients with non-metastatic disease, during the median follow-up for patients alive at the date of censor of 78 months (interquartile range [IQR], 44–114 months), 27 patients (26.7%) and 18 patients (17.8%) experienced progression and any-cause mortality, respectively. In patients with metastatic disease, during the median follow-up for patients alive at the date of censor of 70 months (IQR, 33–112 months), 78 patients (93.9%) and 55 patients (59.1%) experienced progression and any-cause mortality, respectively.

**Table 1:** Clinicopathological characteristics of patients with non-metastatic prostate cancer according to TGFB1 polymorphisms.

Variables	*TGFB1* (rs2241716)			*TGFB1* (rs4803455)		
	CC (n = 44)	CT/TT (n = 57)	P-value	CC (n = 13)	CA/AA (n = 88)	P-value
Median age, years (IQR)	73 (69–77)	71 (65–77)	0.19	70 (61–75)	72 (67–77)	0.31
Median PSA at diagnosis, ng/ml (IQR)	17.3 (8.3–56.1)	9.3 (6.1–31.6)	0.078	12.8 (6.4–91.5)	14.0 (6.6–37.7)	0.96
Biopsy Gleason score, n (%)						
<8	28 (68.3%)	31 (67.4%)		6 (60.0%)	53 (68.8%)	
≥8	13 (31.7%)	15 (32.6%)	0.93	4 (40.0%)	24 (31.2%)	0.58
NA	3	11		3	11	
Clinical T-stage, n (%)						
cT1/2	24 (55.8%)	34 (66.7%)		5 (41.7%)	53 (64.6%)	
cT3/4	19 (44.2%)	17 (33.3%)	0.28	7 (58.3%)	29 (35.4%)	0.13
NA	1	6		1	6	
Clinical N-stage, n (%)						
cN0	39 (88.6%)	50 (89.3%)		10 (76.9%)	79 (90.8%)	
cN1	5 (11.4%)	6 (10.7%)	0.92	3 (23.1%)	8 (9.2%)	0.14
NA	0	1		0	1	
Therapeutic setting, n (%)						
Primary	24 (54.5%)	35 (61.4%)		8 (61.5%)	51 (58.0%)	
Salvage	20 (45.5%)	22 (38.6%)	0.49	5 (38.5%)	37 (42.0%)	0.81
Hormonal therapy						
Combined androgen blockade	16 (36.4%)	21 (36.8%)		5 (38.5%)	32 (36.4%)	
Castration	17 (38.6%)	25 (43.9%)		7 (53.8%)	35 (39.8%)	
Antiandrogen agent	11 (25.0%)	11 (19.3%)	0.77	1 (7.7%)	21 (23.9%)	0.38

IQR, interquartile range; PSA, prostate-specific antigen; NA, not available.

**Table 2:** Clinicopathological characteristics of patients with metastatic prostate cancer according to TGFB1 polymorphisms.

Variable	*TGFB1* (rs2241716)			*TGFB1* (rs4803455)		
	CC (n = 38)	CT/TT (n = 55)	P-value	CC (n = 27)	CA/AA (n = 66)	P-value
Median age, years (IQR)	72 (66–78)	72 (67–76)	0.92	73 (66–77)	72 (67–77)	0.90
Median PSA level at diagnosis, ng/ml (IQR)	144 (62.5–458)	320 (93.4–1400)	0.032*	141 (63.0–566)	294 (87.8–972)	0.21
Biopsy Gleason score, n (%)						
<8	17 (47.2%)	11 (21.6%)		13 (52.0%)	15 (24.2%)	
≥8	19 (52.8%)	40 (78.4%)	0.012*	12 (48.0%)	47 (75.8%)	0.012*
NA	2	4		2	4	
Clinical T-stage, n (%)						
cT1/2	7 (21.9%)	2 (4.3%)		5 (20.8%)	4 (7.3%)	
cT3/4	25 (78.1%)	45 (95.7%)	0.016*	19 (79.2%)	51 (92.7%)	0.081
NA	6	8		3	11	
Clinical N-stage, n (%)						
N0	20 (62.5%)	20 (41.7%)		14 (58.3%)	26 (46.4%)	
N1	12 (37.5%)	28 (58.3%)	0.068	10 (41.7%)	30 (53.6%)	0.33
NA	6	7		3	10	
Hormonal therapy						
Combined androgen blockade	32 (84.2%)	52 (94.5%)		25 (92.6%)	59 (89.4%)	
Castration	6 (15.8%)	3 (5.5%)	0.098	2 (7.4%)	7 (10.6%)	0.64

*Statistically significant. IQR, interquartile range; PSA, prostate-specific antigen; NA, not available.

**Figure 1:**
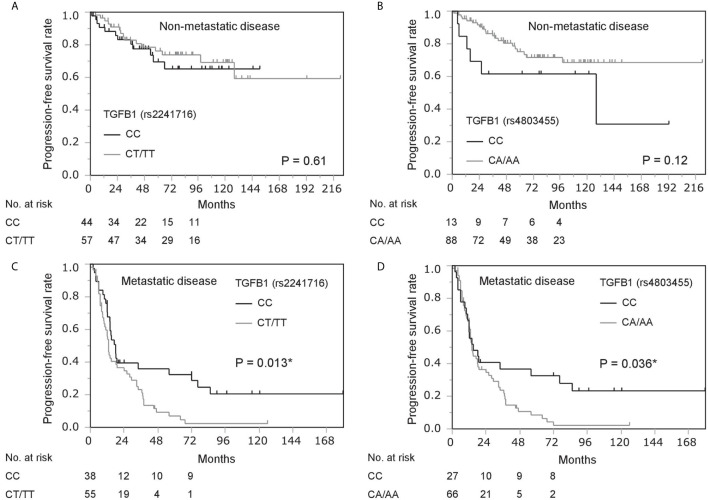
Kaplan–Meier survival analysis of progression-free survival in prostate cancer patients stratified by gene polymorphisms in *TGFB1* (rs2241716 and rs4803455). **(A, B)** Progression-free survival in patients with non-metastatic disease by gene polymorphisms in *TGFB1* [rs2241716 **(A)** and rs4803455 **(B)**]. **(C, D)** Progression-free survival in patients with metastatic disease by gene polymorphisms in *TGFB1* [rs2241716 **(C)** and rs4803455 **(D)**]. *Statistically significant.

We analyzed the association of genetic polymorphisms in *TGFB1* with clinicopathological characteristics and prognosis in patients with non-metastatic disease. Patient backgrounds were comparable in the two subgroups of *TGFB1* genotypes (rs2241716 and rs4803455) in patients with non-metastatic prostate cancer (**Table 1**). No significant association between genetic polymorphisms in *TGFB1* (rs2241716 and rs4803455) and prognosis including progression-free survival (PFS) and overall survival (OS) in patients with non-metastatic disease was observed (**Table 3**, **Supplementary Table 1** and **Figures 1A, B**).

**Table 3:** Progression-free survival according to TGFB1 polymorphisms.

Variable	Non-metastatic disease				Metastatic disease			
	n	HR	95% CI	P-value	n	HR	95% CI	P-value
*TGFB1* (rs2241716)								
CC	44	ref			38	ref		
CT	48	0.78	0.35–1.76	0.56	45	1.85	1.13–3.05	0.015*
TT	9	0.98	0.28–3.51	0.98	10	1.64	0.76–3.56	0.21
Dominant model								
CC	44	ref			38	ref		
CT/TT	57	0.82	0.38–1.76	0.61	55	1.82	1.12–2.94	0.015*
Recessive model								
CC/CT	92	ref			83	ref		
TT	9	1.12	0.33–3.73	0.86	10	1.16	0.57–2.34	0.68
*TGFB1* (rs4803455)								
CC	13	ref			27	ref		
CA	58	0.47	0.18–122	0.12	48	1.71	0.98–2.98	0.059
AA	30	0.54	0.18–1.63	0.28	18	1.87	0.95–3.68	0.069
Dominant model								
CC	13	ref			27	ref		
CA/AA	88	0.49	0.20–1.22	0.13	66	1.75	1.03–2.98	0.040*
Recessive model								
CC/CA	71	ref			75	ref		
AA	30	0.97	0.41–2.31	0.95	18	1.32	0.76–2.28	0.32

*Statistically significant. CI, confidence interval; HR, hazard ratio.

We next analyzed the significance of *TGFB1* genotype among patients with metastatic prostate cancer in the same manner. Analysis of patient backgrounds revealed that PSA value at diagnosis in CT/TT carriers (rs2241716) was higher than that of CC carriers in patients with metastatic disease (**Table 2**). In addition, Gleason score in CT/TT carriers (rs2241716) and CA/AA carriers (rs4803455) was unfavorable compared with that in CC carriers in patients with metastatic disease (**Table 2**). Moreover, clinical T-stage in CT/TT carriers (rs2241716) was more advanced than that of CC carriers in patients with metastatic disease (**Table 2**). Consistent with these findings, the CT/TT alleles in rs2241716 (HR, 1.82; 95% confidence interval [CI], 1.12–2.94; *P* = 0.015) and the CA/AA alleles in rs4803455 (HR, 1.75; 95% CI, 1.03–2.98; *P* = 0.040) were associated with a higher risk of progression during ADT compared with that of the CC allele in patients with metastatic disease (**Table 3**). Similarly, Kaplan–Meier curve showed worse PFS among patients carrying the CT/TT alleles in rs2241716 and the CA/AA alleles in rs4803455 compared with patients carrying the CC allele (**Figures 1C, D**). However, when multivariate analyses incorporating PSA value, Gleason score, clinical T-stage for rs2241716, and Gleason score for rs4803455 were performed, the significance of the CT/TT alleles in rs2241716 (HR, 1.79; 95% CI, 0.98–3.27; *P* = 0.057) and the CA/AA alleles in rs2241716 (HR, 1.34; 95% CI, 0.76–2.35; *P* = 0.31) on PFS diminished. With regard to OS, there was no significant association between the genetic polymorphisms in *TGFB1* and mortality risk in patients with metastatic disease (**Supplementary Table 1**).

Finally, we analyzed the impact of *TGFB1* genotype on survival between patients with non-metastatic and metastatic diseases. Intriguingly, the dominant model of rs4803455 (CC vs. CA/AA; interaction test, P = 0.016) but not the dominant model of rs2241716 (CC vs. CT/TT; Interaction test, *P* = 0.091) was differentially associated with PFS between patients with non-metastatic and metastatic diseases. However, the significance of *TGFB1* genotypes (rs2241716 and rs4803455) on patient backgrounds and OS did not differ between patients with non-metastatic and metastatic diseases (data not shown).

## Discussion

This study showed that genetic polymorphisms in *TGFB1* were associated with unfavorable clinicopathological parameters including PSA value, Gleason score, and clinical T-stage patients with metastatic prostate cancer. Consistent with these associations between *TGFB1* variations and clinicopathological characteristics, the progression risk during ADT was associated with *TGFB1* genotypes, suggesting that *TGFB1* genotypes were associated with PFS through unfavorable tumor characteristics. In addition, *TGFB1* variations were not associated with clinicopathological characteristics and prognosis in patients with non-metastatic disease, and a differential impact of *TGFB1* variation (rs4803455) on PFS between non-metastatic and metastatic disease was observed. Since TGF-β1 has been suggested to play a dual role in the early and later stages of cancer development (11), the differential impact of *TGFB1* genotype on non-metastatic and metastatic diseases may be explained by the distinct biological role of TGF-β signaling according to tumor stage.

A previous study showed that genetic variation in *TGFB1* (509C>T, rs1800469) was associated with Gleason score and tumor stage in prostate cancer (17, 18). Similarly, another genetic polymorphism (*TGFB1*+869T>C, rs1982073) combined with a genetic polymorphism in epidermal growth factor was reported to be associated with time to CRPC (19). Similarly, it has been reported that genetic polymorphism in the promoter region of *TGFBR2* gene coding TGF-βRII was associated with Gleason score and risk of early relapse after ADT among patients with both non-metastatic and metastatic prostate cancer (25). In addition, this study showed that other polymorphisms in *TGFB1* (rs2241716 and rs4803455) were associated with adverse characteristics and progression risk during ADT. These results support the robustness of the association between *TGFB1* genotype and tumor aggressiveness in metastatic prostate cancer, which indicates altered progression risk according to *TGFB1* genotype.

The interactions of TGF-β signaling with EMT and AR signaling may be a possible molecular basis underlying the findings in this study. We previously showed that TGF-β induces AR expression including AR variants through the Twist1 transcription factor, which results in increased EMT phenotype and augmented castration resistance, which is reversed by TGF-β1 inhibitor (13, 14). Therefore, *TGFB1* genotyping may be helpful to identify promising candidates for therapeutics using TGF-β inhibitors, which are under clinical trials (26, 27). As well, TGFB1 genotype could predict durable responders to primary ADT as shown by Kaplan-Meier curve on PFS (**Figures 1C, D**). Although the reason why durable responders carried CC genotype in TGFB1 (rs2241716 and rs4803455), it was suggested that EMT regulated by TGF signaling may play an important role in long-lasting response to ADT.

This study had several limitations. First, this study had a retrospective design. In addition, the study population was limited to Japanese patients, and intensive up-front therapies using docetaxel and novel AR pathway inhibitors were not used at the time of the study. Thus, the significance of *TGFB1* variation in up-front therapies for metastatic hormone-sensitive prostate cancer should be investigated in the future. In addition, the functional effects of the genetic polymorphisms investigated in this study remain unclear. Finally, the correlation between *TGFB1* variation and genetic polymorphism in *TGFBR* or the expression of TGF-β receptor in prostate cancer has not been investigated. Comprehensive investigation on the relationship between TGF-β signaling and ADT would be required in the future.

In conclusion, this study showed that *TGFB1* genetic variations were associated with adverse characteristics and risk of progression during ADT among patients with metastatic disease, but not those with non-metastatic disease. This finding supports a distinct functional role of TGF-β signaling in non-metastatic and metastatic prostate cancer. In addition, *TGFB1* genotyping may be useful to identify candidates for TGF-β signaling–targeting therapies.

## Data Availability Statement

All data relevant to the study are included in the article or uploaded as supplemental information. Deidentified participant data are available upon request.

## Ethics Statement

The studies involving human participants were reviewed and approved by Kyushu University Hospital and University of Occupational and Environmental Health review boards. The patients/participants provided their written informed consent to participate in this study.

## Author Contributions

Conception and design: MS. Acquisition of data: MS, NF, TM, ST, SN, SU, MU, EK, AT, JI, and TU. Analysis and interpretation of data (e.g., statistical analysis): MS and NF. Writing, review, and/or revision of the manuscript: MS and NF. Study supervision: ME. All authors contributed to the article and approved the submitted version.

## Funding

This work was supported by a grant from Takeda Science Foundation.

## Conflict of Interest

The authors declare that the research was conducted in the absence of any commercial or financial relationships that could be construed as a potential conflict of interest.
