# Supplementary material for: Differential Impact of TGFB1 Variation by Metastatic Status in Androgen-Deprivation Therapy for Prostate Cancer
Source: Front Oncol. 2021 May 25;11:697955. doi: 10.3389/fonc.2021.697955 (PMC8186782; doi:10.3389/fonc.2021.697955)
Supplement: Supplementary file 1 [file Table_1.pdf]

Supplementary Table 1. Overall survival according to *TGFB1* polymorphism

	Non-metastatic disease				Metastatic disease			
Variable	n	HR	95% CI	P-value	n	HR	95% CI	P-value
TGFB1 (rs2241716)								
CC	44	ref			38	ref		
CT	48	0.87	0.39–1.96	0.74	45	0.90	0.51–1.57	0.71
TT	9	1.38	0.38–5.00	0.62	10	1.03	0.39–2.72	0.95
Dominant model								
CC	44	ref			38	ref		
CT/TT	57	0.94	0.43–2.03	0.87	55	0.92	0.54–1.57	0.75
Recessive model								
CC/CT	92	ref			83	ref		
TT	9	1.49	0.44–5.01	0.52	10	1.10	0.44–2.75	0.85
TGFB1 (rs4803455)								
CC	13	ref			27	ref		
CA	58	0.51	0.20–1.34	0.17	48	1.31	0.71–2.42	0.39
AA	30	0.82	0.26–2.55	0.73	18	0.88	0.37–2.09	0.78
Dominant model								
CC	13	ref			27	ref		
CA/AA	88	0.58	0.23–1.45	0.24	66	1.19	0.66–2.16	0.56
Recessive model								
CC/CA	71	ref			75	ref		
AA	30	1.38	0.56–3.39	0.48	18	0.74	0.35–1.57	0.43

CI, confidence interval; HR, hazard ratio
